# Neuritic Plaques — Gateways to Understanding Alzheimer’s Disease

**DOI:** 10.1007/s12035-023-03736-7

**Published:** 2023-11-08

**Authors:** Wangchen Tsering, Stefan Prokop

**Affiliations:** 1 Center for Translational Research in Neurodegenerative Disease, University of Florida, Gainesville, FL USA; 2 Department of Neuroscience, University of Florida College of Medicine, Gainesville, FL USA; 3 McKnight Brain Institute, University of Florida, Gainesville, USA; 4 Department of Pathology, University of Florida, Gainesville, USA; 5 Fixel Institute for Neurological Diseases, University of Florida, Gainesville, USA

**Keywords:** Neuritic plaques, Dystrophic neurites, Alzheimer’s disease, Amyloid plaque morphology

## Abstract

Extracellular deposits of amyloid-β (Aβ) in the form of plaques are one of the main pathological hallmarks of Alzheimer’s disease (AD). Over the years, many different Aβ plaque morphologies such as neuritic plaques, dense cored plaques, cotton wool plaques, coarse-grain plaques, and diffuse plaques have been described in AD postmortem brain tissues, but correlation of a given plaque type with AD progression or AD symptoms is not clear. Furthermore, the exact trigger causing the development of one Aβ plaque morphological subtype over the other is still unknown. Here, we review the current knowledge about neuritic plaques, a subset of Aβ plaques surrounded by swollen or dystrophic neurites, which represent the most detrimental and consequential Aβ plaque morphology. Neuritic plaques have been associated with local immune activation, neuronal network dysfunction, and cognitive decline. Given that neuritic plaques are at the interface of Aβ deposition, tau aggregation, and local immune activation, we argue that understanding the exact mechanism of neuritic plaque formation is crucial to develop targeted therapies for AD.

## Introduction

### Pathological Hallmarks of Alzheimer’s Disease

Alzheimer’s disease (AD) is one of the leading causes of death among seniors in the USA. According to data from the Alzheimer’s Association, 1 in 3 seniors is dying of AD or related dementias (RD). Currently, there are over 6 million Americans suffering from AD, and this figure is projected to rise to 13 million by 2050 (Alzheimer’s Association | Alzheimer’s Disease & Dementia Help).

Neuropathologically, AD is characterized by neuronal loss, extracellular amyloid-β (Aβ) deposits in the form of plaques, and intracellular aggregates of tau protein in the form of neurofibrillary tangles (NFT) [1]. Genetic studies strongly suggest that Aβ is at the core of AD pathophysiology. Mutations in genes involved in the proteolytic processes that give rise to Aβ peptides, such as mutations in the Amyloid precursor protein (APP), Presenilin 1(PSEN1), or Presenilin 2 (PSEN2), can cause autosomal dominant AD [2]. APP is a transmembrane glycoprotein that gives rise to Aβ peptides following cleavage by β-secreatase (BACE1) and γ-secretase [3]. PSEN1 and PSEN2 constitute the catalytic subunit of the γ-secretase complex, and AD-associated mutations in these proteins may alter the amount of Aβ produced and/or the ratio of cleavage products [4]. Furthermore, patients with Down’s syndrome (DS, Trisomy 21) harboring three copies of APP are at high risk of developing early-onset AD neuropathologic changes (ADNC). Some DS patients develop diffuse plaques as early as age 12 and almost all the DS patients have Aβ plaque pathology by age 31 [5]. Interestingly, an APP missense mutation (A673T) has been described that decreases APP cleavage by β-secretase and lowers the risk of AD and cognitive decline [6] further supporting the critical role of APP-cleavage in AD pathophysiology.

Evidence from genetic studies led to the formulation of the Amyloid cascade hypothesis (ACH), which postulates that Aβ plaques are the main driver of AD pathophysiology [7, 8]. According to this hypothesis, over-production of Aβ and/or failure of Aβ clearance in sporadic AD (sAD) leads to gradual formation of Aβ oligomers which deposit into Aβ plaques [7]. Aβ oligomers and Aβ plaques eventually cause hyperphosphorylation of the microtubule-associated protein tau which further accelerates widespread neuronal and synaptic dysfunction [7–9]. However, recent failures of clinical trials testing the efficacy of anti-Aβ antibodies put the linearity of the ACH into question. In 2016, Bart De Strooper and Eric Karran expanded the ACH with a more holistic approach by conceptualizing AD into three sequential phases. A biochemical phase of Aβ aggregation and deposition is followed by a cellular phase characterized by activation of the local immune system which further fuels disease progression and Aβ deposition, ultimately culminating in a clinical phase with manifestation of characteristic symptoms [10]. AD is pathologically defined by the presence of extracellular amyloid-β (Aβ) deposits and intracellular aggregates of tau protein [1], but recent studies have demonstrated that a large number of AD cases exhibit a multitude of co-existing pathologies including aggregates of α-synuclein and TDP-43 [11–13]. The role and importance of these co-pathologies in AD is discussed elsewhere [1].

The complexity of AD pathophysiology is further underscored by the fact that the co-existence of Aβ and tau pathology is necessary to fuel the clinical presentation of AD. The presence of either Aβ or tau pathology in isolation is not sufficient to diagnose AD and typically does not result in the clinical picture of AD. Neuropathologically, the presence of only Aβ plaques in the brain has been termed “pathological aging” [14]. Pathological aging patients have mostly diffuse plaques and are often cognitively healthy. Furthermore, the presence of tau aggregates without Aβ plaques in the form of NFT, mostly in the medial temporal lobe, is termed Primary aged-related tauopathy (PART). PART cases are more common among elderly individuals and subjects are generally cognitively unimpaired [15]. It is the interaction and synergy between Aβ plaques and tau proteins, along with the cellular response driven by local glial cells that define AD, and drive neurodegeneration, as well as manifestation of cognitive symptoms.

Understanding the interaction and synergy between Aβ and tau has long been the holy grail of AD research. Both protein pathologies are initially observed in anatomically distinct regions and only later in disease course converge. Thal and colleagues documented the spread of Aβ plaques through the brain in five different stages [16]. The first Aβ plaque deposits are seen in the neocortex and then spread into limbic regions, basal ganglia, and thalamus, and eventually to the brainstem and cerebellar cortex. On the other hand, NFT pathology spreads through the brain following a distinct sequence. Braak and Braak showed that NFT initially aggregate in the entorhinal cortex and then spreads into the hippocampus, eventually, spreading into neocortical areas [17]. A later study from Braak and colleagues revised the staging scheme and showed that early tau hyperphosphorylation can be detected in the lower brainstem (locus coeruleus) even before any tau pathology is noted in the transentorhinal region [18]. How do Aβ and tau interact when the pathologies appear to originate in anatomically different brain regions? There are a few possible hypotheses of how Aβ and tau interact at a distance, but it may be more informative to look at instances when these two protein pathologies are in close vicinity to each other. One very prominent example are neuritic plaques (NP), a subset of Aβ plaques surrounded by swollen or dystrophic neurites (DN). Most NP are associated with tau-positive DN and are also accompanied by a strong activation of the local immune system, in particular microglia. In the following section, we will discuss different morphologies of Aβ plaques to highlight the uniqueness of NP, describe current histological and immunohistochemical markers for NP, and discuss prevailing theories of NP formation.

### Morphology of Aβ Plaques and Introduction to Neuritic Plaques

Aβ plaque is a common term used to describe Aβ depositions in tissues. Aβ deposits can be detected using different staining methods. Some Aβ plaques are positive for Congo red and are therefore termed “congophilic plaques,” while Aβ plaques detected by silver-based staining are termed “argyrophilic plaques.” Immunohistochemical staining with anti-Aβ antibodies is currently the most widespread used method to detect these Aβ deposits. Based on different staining properties, a plethora of Aβ plaque morphologies have been described in the literature, including but not limited to “dense-core,” “burned-out,” “cotton-wool” [19], “coarse-grain” [20], and “bird nest” [21]. Dickson and Vickers categorized Aβ plaque types as diffuse, fibrillar, or dense-cored using Thioflavin S staining [22]. More broadly, Aβ plaque can be morphologically classified as either “densed-core plaque” or “diffuse plaque.” Densed-core plaques typically have congophilic cores, so other morphological structures such as “compact,” “classical,” “mature,” and “coarse-grain” [20] could be grouped under this broad category. Diffuse plaques are typically negative for Congo red and have very loose shapes and sizes and include “Cotton-wool plaque” [19] and “lake-like plaques” [23]. Not only are some of these morphologies only seen in a subset of AD cases, even within a given patient brain plaque morphologies can vary widely between brain regions. For example, the cerebellum in AD cases predominantly shows diffuse plaques [24].

**Fig. 1:**
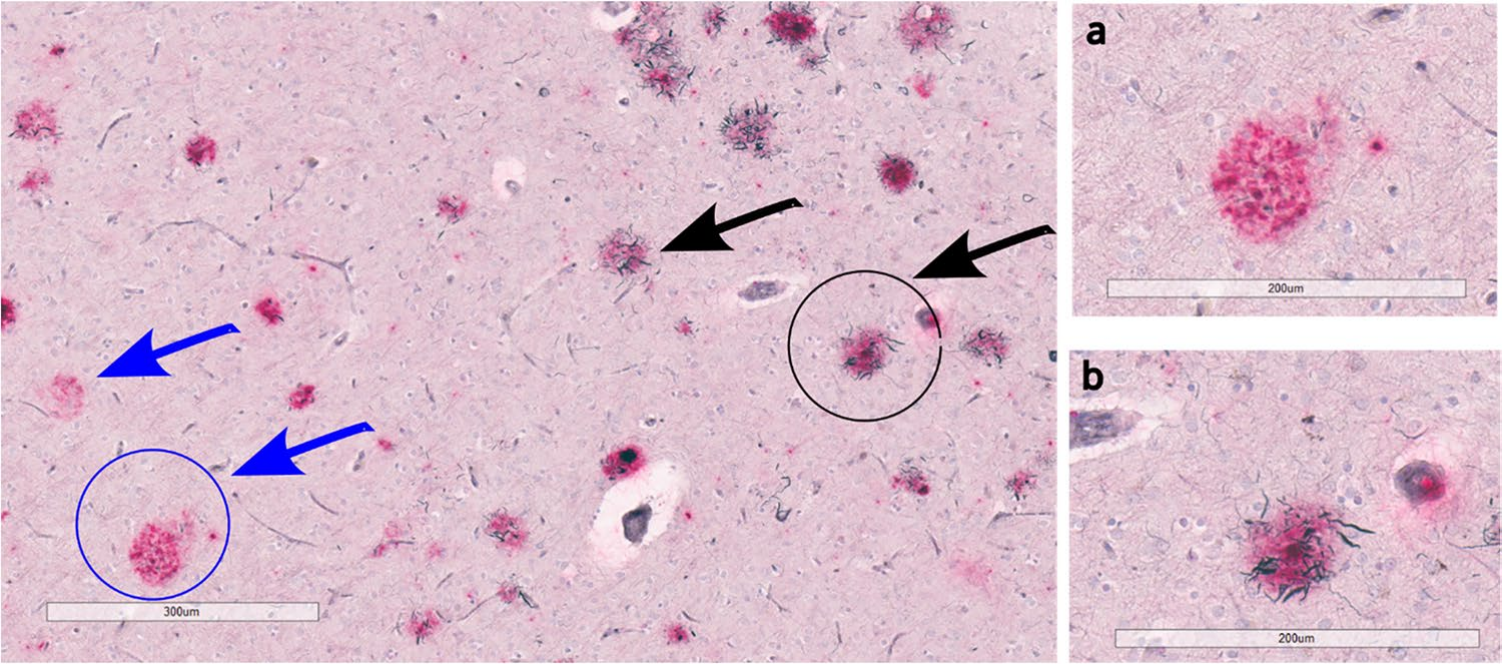
Neuritic plaques (black arrows) and non-neuritic plaques (blue arrow) in the cortex of an Alzheimer’s disease postmortem brain tissue, stained with Gallyas Silver staining (black color) and Aβ antibody antibody (Ab5, Pink color). **a** 20× image of non-neuritic plaque. **b** 20× image of neuritic plaque

In 1970s, Henryk Wisniewski and Robert Terry found that DN are a major constituent of Aβ plaques and coined the term “neuritic plaque,” NP [25]. NP are a subset of Aβ plaques that are associated with DN, swollen neurites originated mostly from axons [26–29] (Fig. 1). DN can also originate from dendrites, but this rarely happens in AD [29]. Dickson and Vickers showed that DN can be associated with both diffuse plaques and dense-cored plaques with approximately 80% of DN associated with dense-core plaques in end-stage AD cases, as compared to only 20% association with diffuse plaques [22]. NP are arguably the most significant and important morphological subtype of Aβ plaques because swollen neurites associated with NP suggest neuronal insult and tissue damage [30].

The exact trigger causing the development of one morphological subtype over the other is still unknown. However, there is the notion that NP are evolving from diffuse plaques [31].

Diffuse plaques transform into NP when the Aβ deposition occurs in brain areas with neurites vulnerable to paired helical filament-related degeneration. In brain regions with no vulnerable neurites, diffuse plaques remain diffuse and do not evolve into NP [32]. Evidence for this transformation of plaque types was provided by two-photon imaging studies in animal models which demonstrated that Aβ deposition preceded and caused selective formation of neuritic dystrophies [33, 34]. However, other studies in animal models inoculated with distinct Aβ seeds indicate that different conformers of Aβ induce specific Aβ plaque morphologies [35–37], suggesting that different types of Aβ deposits develop and evolve independently. More recent large-scale proteomic studies have fueled speculations that Aβ deposits provide a scaffold for other proteins to co-accumulate with plaques during AD progression and these plaque-associated proteins are in turn thought to be responsible for inducing neuritic dystrophy and neurodegeneration [38]. These examples illustrate that the exact mechanism of how diffuse plaques and NP develop in AD is still the subject of much discussion and controversy.

### NP and Its Correlation with Network Dysfunction and Cognition

DN are swollen and altered axonal structures filled with many different vesicles and organelles such as mitochondria [39]. Axons are important for the transport of neuronal cargos as well as for relaying information between neurons. Axonal alteration and degeneration are often seen in many different neurodegenerative diseases. For example, DN are found in patients with traumatic brain injury [40], cerebral stroke [41], and cortical dysplasia [42]. In AD however, DN are almost always associated with Aβ plaques in the form of NP.

DN around Aβ plaques have been traced temporally in APP/PS1 mouse model using two-photon imaging and are found to be highly dynamic and continuously remodeled over time [33]. In TgCTRND8, an Aβ plaque mouse model, even extreme neuritic dystrophy, can maintain continuity for a long time [43]. DN formation and axonal alterations are a slow process and have been shown to disrupt the neural network for a long time [44, 45].

A recent study in 5xFAD mice suggests that hundreds of axons become dystrophic and swollen around a single Aβ plaque, and that DN around the Aβ plaque act as an electric current sink [46]. By measuring calcium ion influx between and within the mouse brain hemispheres, the authors found that axonal swellings reduce and sometimes block the action potential propagation [46]. The blockage of action potential conduction by DN disrupts long-range connectivity in the brain, leading to whole-brain neural network dysfunction over time. In postmortem human brains, the brains from patients with AD have more average DN per Aβ plaque and have larger DN compared to those from patients with mild cognitive impairment (MCI) [46]. Since the presence of DN correlates well with cognitive impairment, it would be interesting to see whether cognitive impairment in AD is largely driven by disruption in long-range connectivity or tau-mediated neuronal loss.

Studying the correlation between Aβ plaque deposition and cognition is complicated by the different morphologies of Aβ plaques. Diffuse plaques are often abundant in cognitively healthy older people (pathological aging) without tauopathy [14]. Therefore, diffuse plaques are considered benign and are not associated with significant clustering of microglia. On the other hand, NP have been shown to have a higher correlation with cognitive decline than diffuse plaques [47]. In fact, a study of 123 non-cognitively impaired older participants from the Rush Religious Order Study showed that the presence of NP was associated with lower performance in the cognitive domains such as episodic and semantic memory, as compared to the presence of diffuse plaque, after adjusting for APOE carrier status, age, and gender [48]. The higher correlation of cognitive impairment with NP is rather not surprising because NP contains p-tau-associated neurites and it has been shown that tau pathology burden has a higher correlation with the severity of cognitive impairment observed in AD [49]. In a study of 334 autopsied subjects, both medial temporal NFT and isocortical NP contributed significantly to cognitive impairment [50].

### Association of NP with Local Immune Activation

**Fig. 2:**
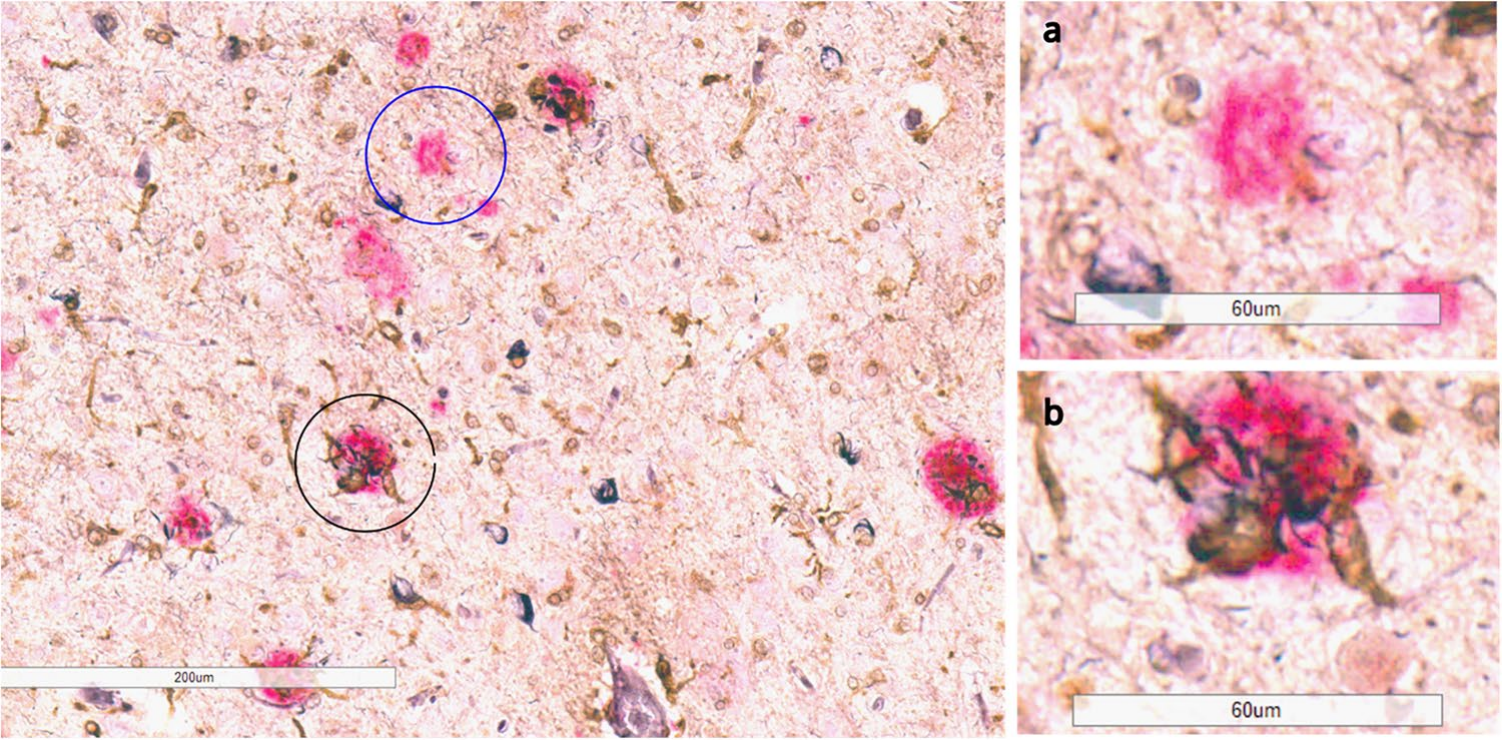
Microglia cluster around neuritic plaques (black circle) and not around non-neuritic plaques (blue circle) in the cortex of an Alzheimer’s disease patient, stained with Gallyas Silver staining (black color), Aβ antibody (Ab5, pink color), and microglia marker (ferritin, brown). **a** 40× image of ferritin-negative non-neuritic plaque. **b** 40× image of ferritin-positive neuritic plaque

Neuroinflammation mostly mediated by microglia activation is increasingly becoming an imperative facet of AD, along with Aβ plaques and NFT [51]. In fact, a human positron emission tomography brain imaging study showed that the presence of Aβ, tau, and microglia together have the highest correlation with cognitive impairment, as compared to the presence of any single pathology or Aβ and tau together [52]. Although the role of microglia in AD pathophysiology is still elusive, there is growing consensus that microglia are more clustered and activated around NP compared to diffuse plaques (Fig. 2) [53–60]. When microglia are ablated by the application of CSF1R inhibitors in murine model systems, DN formation around the Aβ plaques is reduced [57]. Furthermore, mice that lack microglia from birth exhibit mostly diffuse plaques and lack NP formation [58] suggesting that the presence of microglia is also necessary for DN formation around Aβ plaques.

Microglia have many receptors to respond to and phagocytose pathogens and foreign bodies. Triggering receptor expressed on myeloid cells 2 (TREM2) is a protein that is expressed on microglia and is important for microglial phagocytosis of Aβ plaques. Mutations in the TREM2 gene have been associated with an increased risk of developing AD. Studies in postmortem brain tissue as well as animal studies have shown that the loss of TREM2 function significantly increases p-tau-associated dystrophic neurites around Aβ plaques as well as overall tau burden [59, 60]. Interestingly, chronically increasing TREM2 signaling also significantly increases p-tau-associated DN in a mouse model [61]. Therefore, the TREM2-mediated effect on p-tau-associated DN depends on the context and stage of the disease.

Why and how microgliosis is targeting NP is still not known. Some researchers suggest that NP are the end-product of neuronal cell lysis [62, 63]. When neurons burst and leak their cytoplasmic contents, microglia cluster and form NP. Other researchers suggest that Aβ oligomers are neurotoxic and that Aβ oligomers on the extracellular Aβ plaques cause axonal dystrophy, which leads to microglial activation around Aβ plaques. In a recent study, Piezo1, a mechanoreceptor on microglia, is shown to sense Aβ fibril stiffness [64]. The activation of Piezo1 triggers calcium influx, which leads to microglial clustering and phagocytosis of Aβ plaques. It is possible that DN around Aβ plaques make the plaques stiff, which then activates Piezo1 and induces microglial clustering.

Aside from microglia, astrocytes are another important mediator of neuroinflammation in AD [65] and reactive astrocytes also surround NP [66–68] but their contribution to NP formation and maintenance is less studied to date.

### NP as the Key Interface Between Aβ and Tau in AD

It has been shown that Aβ can drive tau alterations in both animal models as well as in cell culture experiments. However, how Aβ drives and accelerates tauopathy is still elusive. One possible mechanism that links Aβ and tau is the DN. DN serve as a microenvironment where Aβ, tau and microglia interact. In a study from Virginia Lee’s group, human brain-derived pathological tau (AD-tau) was injected into two Aβ mouse models, 5xFAD, and APP KI mice which express human APP carrying the Swedish double mutation under control of the murine APP promotor at different stages of Aβ deposition [69]. AD-tau injection into the hippocampi and underlying cortex of 5xFAD mice exclusively showed AT8-positive DN around Aβ deposits with very little development of NFT pathology. However, when AD-tau was injected into wild-type mice, which do not exhibit Aβ plaque pathology, NFT-like aggregates, but not AT8-positive DN were noted, suggesting that DN formation required interaction between Aβ plaques and pathological tau. AD-tau injected 5xFAD mice induce very little NFT pathology as compared to NP tau. When the authors injected a higher amount (8 ug) of AD-tau into 4-month-old 5xFAD mice, it induced more NFT than in 5xFAD mice that were injected with only 4 ug of AD-tau, suggesting that AD-tau caught up in DN seeded NFT in the soma. This observation suggests that DN are the site where Aβ plaque facilitates tau accumulation, which eventually leads to the formation of NFT [69]. Whether or not NP are sufficient to drive the pathological conversion of tau into NFT remains elusive. Tong Li et al. showed in mouse models that NP are necessary but not sufficient to convert wild-type tau into pathological tau. They suggested that along with NP, a second risk factor such as APOE4 is required to facilitate the conversion of wild-type tau into pathological tau [70].

## NP — Detection Methods, Markers, and Temporal Appearance

DN associated with Aβ plaques were first discovered by using conventional staining methods such as Gallyas or Bielschowsky silver staining. Over time, the sensitivity of immunohistochemistry (IHC) led to the visualization of DN using many antibodies against different cellular organelles that are trapped within DN. It has been shown that some of the axonally transported proteins such as APP [71, 72], BACE1 [73–76], and LAMP1 [77] are part of DN. However, the precise role and mechanistic implications of different DN markers in the pathogenesis of Aβ plaques is still unknown. Some researchers broadly categorize DN as either dystrophic-type neurites or PHF-type neurites [78]. Dystrophic-type neurites contain markers of lysosomes, autophagy, and APP and are associated not only with AD but also with aging. Elderly non-demented subjects can have APP-positive DN in the neocortex and hippocampus but lack tau-positive DN [72, 79]. However, PHF-type neurites typically contain tau protein [80] and are shown to be specific and unique to AD. Interestingly, LAMP1, APP, and BACE1 DN have weak co-localization with tau-positive DN [74, 81]. This weak colocalization suggests that either there is a distinct spatial event in DN formation or two independent types of DN exist without correlation [82].

It is difficult to track an individual Aβ plaque spatially and temporally to understand the sequential formation of DN. In mouse models, it has been shown that DN form sequentially in different layers during Aβ plaque growth. Early autophagy and lysosomal proteins form the initial layer of DN followed by tubular ER proteins and then late autophagy/endosomal proteins such as LC3 [83, 84]. In general, studies in both mouse models and human postmortem brains showed that both APP immunoreactive and ubiquitin immunoreactive DN appear early during Aβ plaque formation, while PHF/tau DN appear slowly and late, suggesting a temporal sequence of DN marker appearance during AD progression [80, 85–89] (see also Table 1). This sequence of events in the formation of DN is extrapolated from different brains at different time points (cross-sectional studies). Therefore, a technique to track the same individual DN maker such as LAMP1 or APP in a single Aβ plaque over time would be informative and necessary to understand the exact sequence of DN formation.

**Table 1:** Summary of dystrophic neurite (DN) markers and their temporal appearance in AD progression

DN markers	Pathways	Temporal appearance in AD progression	References
APP	Amyloidogenic	Early	[71, 72, 80, 85]
BACE1	Amyloidogenic	Early	[73–76]
LAMP1	Lysosomal	Early	[77–82]
RTN3	Endoplasmic reticulon	Early	[83]
SAP-C	Lysosomal	Early	[84]
Ubiquitin	Degradation	Early/intermediate	[85]
Neurofilament	Neuronal cytoskeleton	Early/intermediate	[80, 86, 90]
p-tau	Microtubule stabilization	Late	[80, 86, 87, 90]

**Fig. 3:**
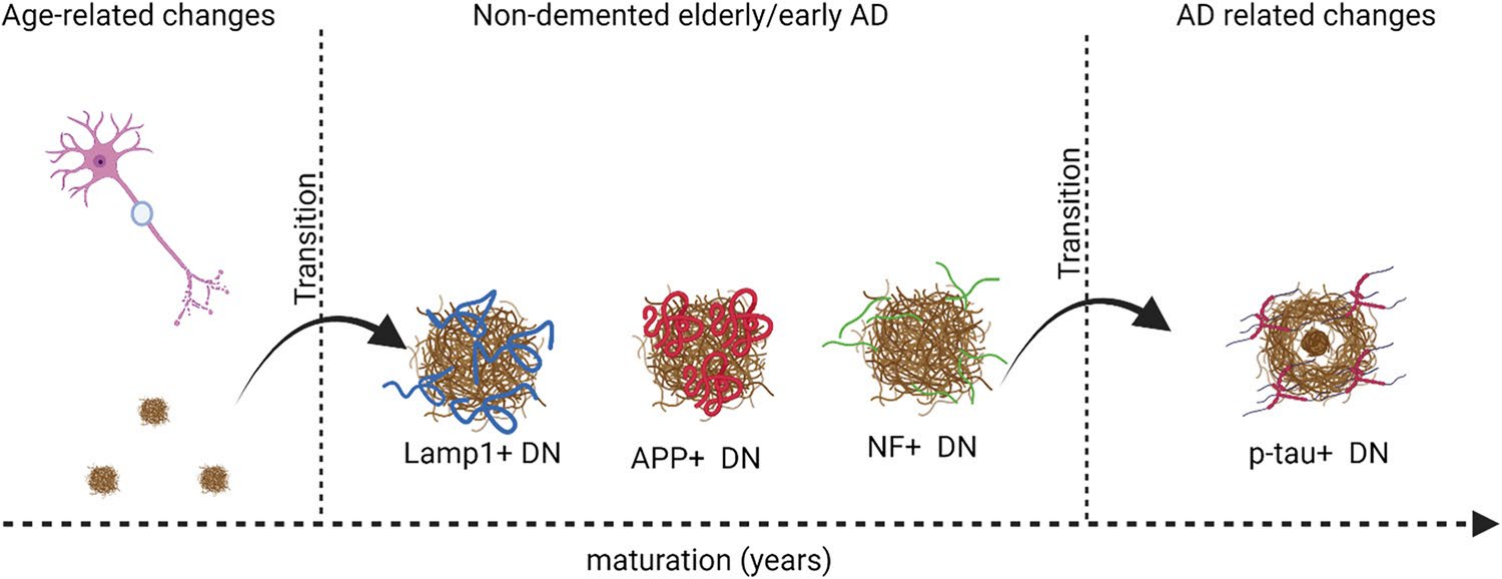
DN evolve over time during AD progression. Independent of amyloid plaques, DN can be associated with aging. Once amyloid plaques are developed, APP+/Lamp1+ DN appears first, followed by NF+DN, and lastly p-tau+ DN. p-tau+ DN are associated with cognitive decline. Created by BioRender.com

Aging is a major risk factor for neurodegenerative diseases such as AD. The molecular and cellular changes in normal aging and age-related diseases share similar signatures. Aβ deposition in the form of diffuse plaques (pathological aging) is associated with aging. Interestingly, DN are observed in human and AD-mouse brains prior to Aβ deposition [91, 92] suggesting that early DN formation is part of normal aging. As Aβ plaques start to develop, they disrupt the axonal structure and cause DN formation, marked by positivity for APP, Lamp1, and ubiquitin. Over time, during the Aβ deposition, axonal dystrophy transforms into AD-related DN marked by the presence of PHF/tau. PHF/tau DN seems to represent the symptomatic and end-stage AD (Fig. 3).

## Mechanisms of NP Formation

There are multiple hypotheses and concepts about how DN and NP are formed in AD. In the following sections, we will discuss the prevailing theories of NP formation.

### Extracellular Aβ Causes Dystrophic Neurites

One prevailing hypothesis suggests that extracellular Aβ is neurotoxic and induces microtubule disruption, impaired axonal transport, and causes axonal dystrophy around Aβ plaques [7, 75]. This hypothesis assumes that Aβ plaque deposition occurs before DN formation. Studies on primary neurons have shown that an application of Aβ42 oligomers results in beaded neurites with disruption in microtubules and axonal transport [75, 93]. Similarly, treating cultured human-induced pluripotent stem cell (iPSC)-derived neurons with synthetic Aβ42 causes p-tau-positive axonal swellings surrounding Aβ plaques [94]. Aβ oligomers derived from AD brains cause neuritic dystrophy and AD-type tau alterations, indicating that the neurotoxicity of Aβ oligomers causes axonal dystrophy (ND) in cell culture [95, 96]. Although most experiments demonstrating the direct neurotoxic effects of Aβ are performed in cell culture systems, there are a few in vivo studies that have also demonstrated the direct neurotoxic effect of Aβ plaques on surrounding neurites in the form of neuritic dystrophy by using multiphoton microscopy [97].

The neurotoxic effect of extracellular Aβ is suggested to be mediated by calcium dyshomeostasis. Studies have shown that Aβ exposure to neurons elevates intracellular calcium levels in different model systems [98, 99]. It has been suggested that Aβ plaques can induce loss of membrane integrity through different mechanisms, such as forming pores in the membrane, resulting in an influx of calcium [100]. An uncontrolled influx of calcium ions can disrupt the ionic gradients necessary to maintain neuronal activity and neurotransmitter release. Interestingly, blocking Aβ and calcium-dependent mechanisms, such as calcineurin (CaN) activation, have been shown to prevent Aβ-induced axonal dystrophy [101]. According to the ACH, Aβ oligomers gradually deposit as Aβ plaques and then alter tau activities, leading to neuronal dysfunction [7].

It is unclear whether axonal dystrophy is caused by the neurotoxicity of Aβ considering most experiments were done by exogenous application of Aβ aggregates in cell culture. Aβ oligomer studies often face criticism for using high concentrations of Aβ oligomers in cell culture that are not physiologically relevant and unlikely to occur in vivo. Moreover, most animal models of Aβ plaque formation do not induce tauopathy and downstream neurodegeneration. Furthermore, if Aβ is directly neurotoxic, why does Aβ in diffuse plaques fail to elicit harmful responses, whereas NP are associated with neurodegenerative changes? Why is there a long delay, or silent phase, from Aβ deposition to neurodegeneration? These and many other questions surrounding the direct neurotoxicity of Aβ aggregates prompted the search for alternative explanations.

One alternative explanation proposed by Todd Golde [38] is that Aβ aggregates are not directly neurotoxic but serve as a scaffold for other molecules or proteins that drive neurotoxicity-mediated neurodegeneration [32]. Proteomic and transcriptomic analysis of postmortem brain tissue reveals that there are many bioactive signaling molecules that co-accumulate within Aβ plaques in AD [102–104]. It is possible that extracellular Aβ plaques serve as a scaffold for signaling molecules that co-accumulate within Aβ plaques and cause presynaptic axonal dystrophy. This notion assumes that Aβ plaques are necessary but not sufficient to cause DN formation and downstream neurodegeneration without certain associated signaling molecules (Amyloidosis-associated proteins, AAP) [38].

Another cause of axonal dystrophy around Aβ plaques could be the physical growth of Aβ plaques, not the toxicity of Aβ. When Aβ plaques grow over time, they disrupt the axons of nearby neurons [86, 105, 106]. The physical push and disruption of axons by Aβ plaques can cause axonal dystrophy. However, there is no correlation between the size of Aβ plaques and axonal dystrophy, disputing the notion that the physical growth of Aβ plaques is the only cause of axonal dystrophy.

### Intracellular Aβ Causes Dystrophic Neurites via Autophagy and Lysosomal Disruption

Another hypothesis about DN formation is that intracellular Aβ causes axonal dystrophy through autophagic and lysosomal processes. Intraneuronal Aβ42 has been shown to accumulate in neurons in both cell culture and mouse models [107–109]. It is unclear whether Aβ/APP is formed intracellularly during APP processing or through the uptake of extracellular oligomeric Aβ seeds. Regardless of the origin of perinuclear Aβ/APP fragments, these misfolded Aβ/APP fragments are shown to accumulate in lysosomal and autophagic vesicles [62, 110]. In different Aβ plaque transgenic mouse models, poorly acidified autolysosome buildup containing Aβ/APP fragments distorts the plasma membrane and forms a flower-like dystrophy, which the authors called PANTHOS (P=poison, ANTHOS = flower) [110]. PANTHOS is described as the result of autolysosomal failure to digest and break down Aβ/APP fragments and other cellular cargos. When poorly acidified autolysosomes become too big and swollen, neurons die and PANTHOS transforms into an NP [110]. In both human brain and mouse models, nuclear remnants were found at the center of the NP using the neuronal nuclear marker, NeuN [62, 63], suggesting that each NP is the end product of neuronal cell lysis. However, in mouse models such as 5xFAD, neuronal loss is not found [111], which begs the question of whether PANTHOS is the main driver of AD pathology or is localized and has no significant role in AD pathophysiology.

The intracellular Aβ hypothesis of NP formation suggests that extracellular Aβ plaques are the tombstones of intracellular Aβ accumulation and autolysosomal defects in AD. Thus, arguing in favor of autolysosomal and autophagic defects as a cause of AD pathology rather than consequences of Aβ plaque deposition. Autolysosomal and autophagic defects can be attributable to mutations in Presenilin. A recent study on iPSC human neurons demonstrated that inhibition of γ-secretase results in lysosomal and autophagy dysfunction such as elevated levels of LAMP1 and LC3, suggesting that intraneuronal accumulation of APP-CTF99 has an undesirable effect on neurons [112]. Intraneuronal accumulation of APP-CTF99 also leads to endosomal-autophagic-lysosomal dysfunction in in vivo models [113, 114]. Under normal conditions, Presenilin 1 (PSEN1) helps acidify lysosomes. Loss of PSEN1 function disrupts acidification and proteolysis of lysosomes, which eventually inhibits autophagy [115, 116]. The inhibition of lysosomal proteolysis due to mutations in PSEN1 stalls the axonal transport of autophagic vacuoles and late endosomes, forming DN [117]. In cell culture, PSEN1 knockout (KO) neurons exhibit a higher frequency of DN compared to control. When PSEN1 KO neurons were treated with TRPML1 blocker Ned-19, the frequency of DN decreased to a level comparable to controls [115]. Similarly, pharmacologic agents that disrupt lysosomal proteolysis slow the lysosome movement along the axon and cause axonal dystrophy in primary neurons [118]. The axonal dystrophy and movement recovered after lysosomal proteolysis were restored [118], suggesting that lysosomal dysfunction can contribute to axonal dystrophy in the absence of Aβ/APP fragments in cell culture.

Although the intracellular Aβ hypothesis of NP formation is intriguing, it is still largely restricted to artificial systems such as transgenic mouse models and cell culture systems. Without substantial evidence in human AD cases, it is difficult to ascertain whether the accumulation of intracellular Aβ and autolysosomal defects are artifacts of genetic overexpression in a non-relevant model system or an AD-related phenomenon.

### Ferroptosis — p-Tau+ Droplet Degeneration Leads to NP Formation

Iron deposition is often associated with aging and neurodegenerative diseases including AD [119–121]. High levels of brain iron are toxic to neurons and microglia [122, 123]. Streit et al. proposed that NP are formed by Aβ encasement of p-tau+ degeneration droplets that are caused by iron overload [124]. Based on Streit et al., p-tau+ pretangle neurons face two fates: either form an intracellular filamentous inclusion that develops into NFT or form an extracellular droplet sphere that transforms into NP. This hypothesis suggests that p-tau+ droplet spheres mark neuronal dissolution due to ferroptosis, which occurs in the absence of Aβ deposition [124]. Ferroptosis is a type of cell death caused by iron overload in the brain. When a neuron dies due to ferroptosis, it releases the p-tau and iron into the extracellular space, which is then contained and encased by Aβ, leading to NP formation. This hypothesis of NP formation suggests that Aβ deposition is a protective mechanism to limit the extracellular spread of free iron [124]. Intracerebral co-injection of iron and Aβ in rats significantly increased neuronal loss as compared to injections of Aβ alone [125], indicating that iron mediates the neurotoxicity associated with amyloid plaques. Interestingly however, co-injection of iron and Aβ was significantly less toxic than injection of iron alone, hinting that Aβ deposition may be a mechanism to protect the brain from iron toxicity. To support this hypothesis of NP formation from p-tau+ pretangles, a recent differential protein expression study using the Digital Spatial Profiling (DSP) technique found upregulation of Aβ processing proteins such as APP and BACE1 in NFT-bearing neurons as compared to non-NFT-bearing neurons [126].

The p-tau+ droplet degeneration hypothesis of NP formation is based on cross-sectional studies of human postmortem brain tissues and lacks detailed mechanistic studies. Moreover, if Aβ deposition is a protective mechanism to limit p-tau+ droplet degeneration, why don’t tau mouse models such as PS19 or AD-tau injected wild-type mice develop Aβ deposition? Aβ deposition is not seen in tauopathies such as progressive supranuclear palsy (PSP), corticobasal degeneration (CBD), Pick’s disease (PiD), and primary age-related tauopathy (PART) as a protective mechanism to limit tau. Streit et al. showed that iron deposition is localized in the core of Aβ plaques using Prussian blue staining, arguing that Aβ deposition is encasing the iron [124]. However, the temporal sequence of Aβ encasement of iron is still unknown. Iron deposition is also a characteristic feature of microhemorrhages. It has been shown that NP are confined to the pericapillary area and associated with microvasculature degeneration [127], which could be an alternative explanation for the propensity of NP to be positive for iron.

Furthermore, Munoz and Wang observed aggregates of DN associated with NFT in the absence of Aβ plaques, which they termed tangle-associated neuritic clusters (TANCs) [128]. TANC is mostly abundant in the hippocampus of AD patients. Are TANCs the same as p-tau+ droplet spheres as well? The relationship between TANC and p-tau+ droplet spheres is still unknown. The abundance of p-tau+ droplet spheres and TANC in the hippocampal subregions could imply that NP formation in the hippocampus follows a different mechanism than NP formation in cortical regions.

## Conclusion

NP are a unique feature of AD. Numerous hypotheses and ideas exist regarding the formation of NP in AD. The two major hypotheses are that (1) extracellular Aβ causes axonal dystrophy, and (2) autolysosomal packaging of intracellular Aβ results in dystrophy and neuronal death. Can these two major hypotheses exist together or oppose each other? If these two hypotheses can exist together, how do they complement or synergize?

AD is complex and can be viewed as a brain organ failure [38]. It is entirely possible for different mechanisms to occur simultaneously or sequentially in the same or different brain regions. One possible mechanism is that local differences in APP processing lead to the generation of different types of NP in a brain region-specific manner.

**Fig. 4:**
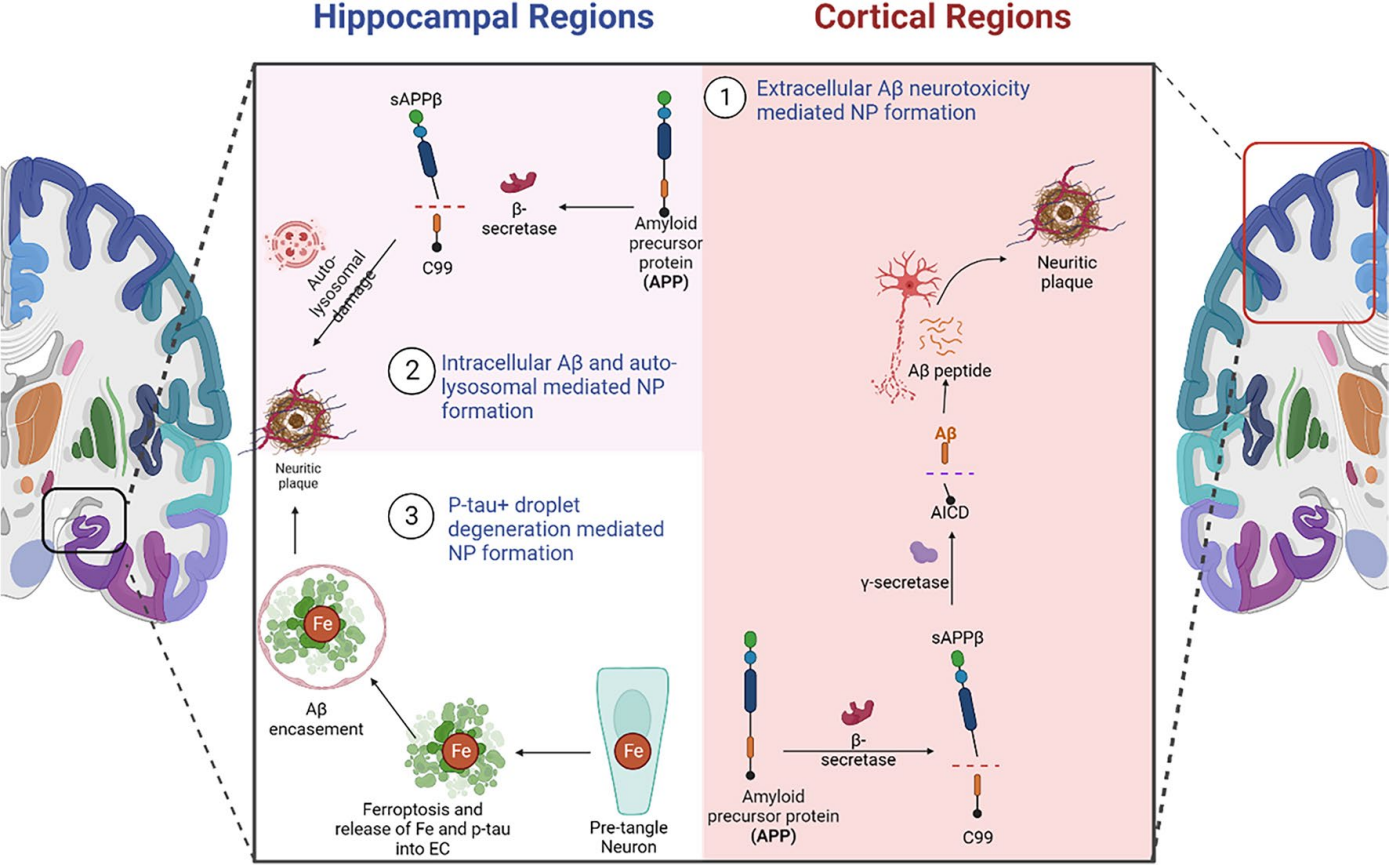
Independent NP formation mechanisms in different brain regions. Created with BioRender.com

Here we speculate that independent and distinct mechanisms of NP formation exist in the hippocampus and neocortex (Fig. 4). Intracellular Aβ-mediated NP formation (PANTHOS) and p-tau+ droplet degeneration-mediated NP formation seem to be unique to the hippocampus and subiculum. On the other hand, extracellular Aβ-mediated NP formation is prominent in cortical regions but can be seen in the hippocampus as well. The hippocampus is one of the brain regions that develop AT8+ tau pathology first in AD, even before the appearance of Aβ pathology [129]. Therefore, it is reasonable to speculate that NP mechanisms unique to the hippocampus (PANTHOS, p-tau+ droplet degeneration) evolve first during AD progression, either as an aging phenomenon or a disease-relevant phenomenon. The temporal sequence of NP formation mechanisms is unclear and needs to be investigated in the future.

Despite an ongoing discussion about the precise mechanism underlying NP formation, it is evident that NP are a key pathological hallmark of AD. NP have a higher correlation with cognitive decline, astro- and microgliosis, higher chances of long-range neuronal network disruption, and higher interaction with p-tau as compared to non-neuritic plaques (Table 2). Aβ plaques and NFT in isolation have been described in conditions such as pathological aging and PART respectively, but the interaction of Aβ and tau, associated with the formation of NP, seems to be the defining feature of AD.

**Table 2:**
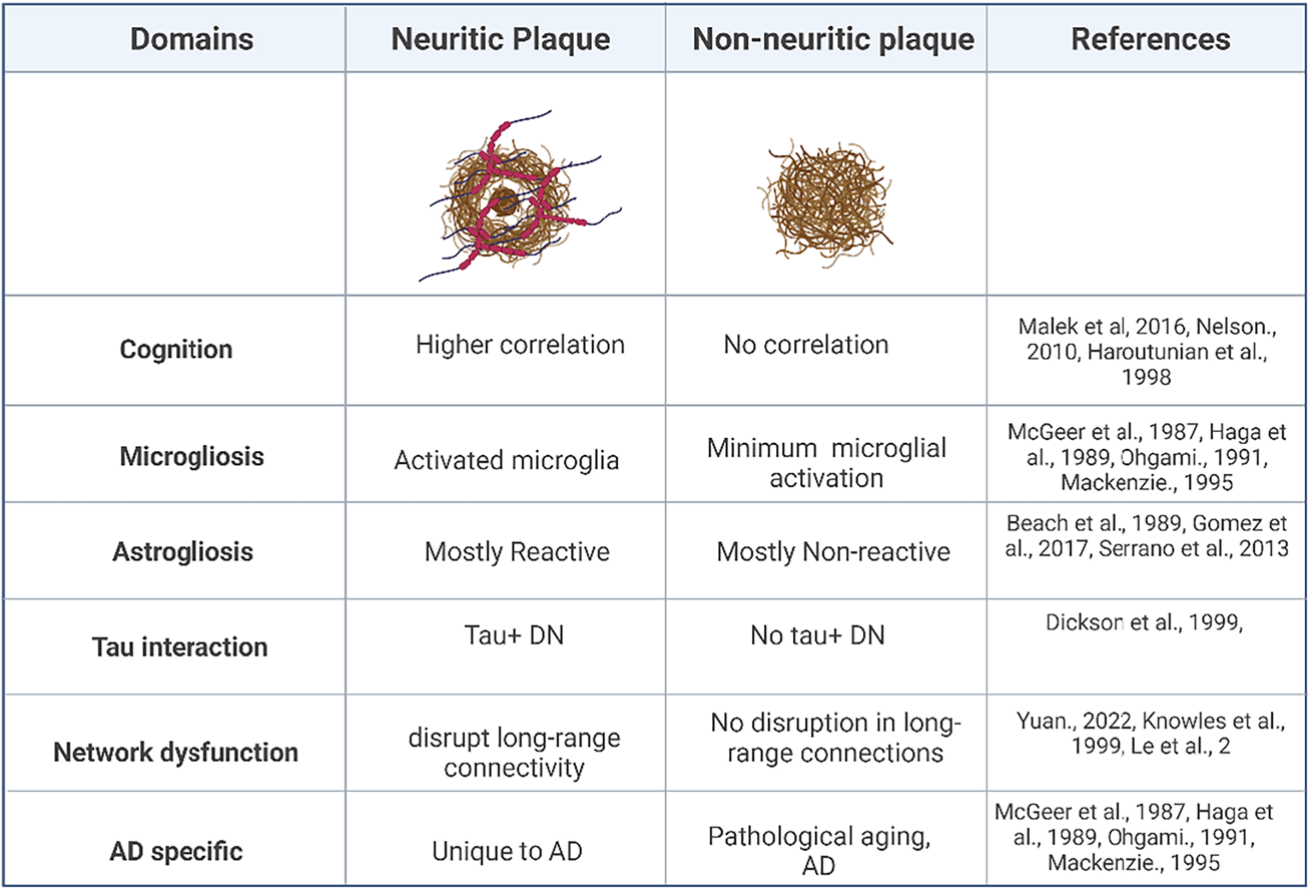
Summary illustrating the role of neuritic plaque and non-neuritic plaques in AD pathophysiology. Created with BioRender.com

From a therapeutic point of view, studies in animal models suggest that the process of NP formation can be halted or even reversed through targeted interventions. For example, Holtzman and colleagues have shown that passive immunization with anti-Abeta antibodies can reduce and recover neuritic dystrophy in PDAPP transgenic mice [34]. Other studies have demonstrated that neuritic dystrophy can be remedied in animal models at least in the early stages of DN formation [33]. Therefore, any kind of intervention in the early stages of DN formation might be the key to halt further progression of neurodegenerative cascades. Based on the proposed mechanisms of NP formation discussed above, different therapeutic strategies can be envisioned such as enhancing autolysosomal function, reducing iron overload, targeting co-accumulating proteins, or preventing the transformation of diffuse plaques into NP. It will be very important moving forward to get a better understanding of mechanisms underlying NP formation to refine therapeutic interventions to halt the pathophysiological cascade leading to cognitive decline and dementia in AD.

## Data Availability

Not applicable.

## Abbreviations

*AD*: Alzheimer’s disease
*AAP*: Amyloidosis-associated proteins
*APP*: Amyloid precursor protein
*BACE1*: β-secretase
*ACH*: Amyloid cascade hypothesis
*DN*: Dystrophic neurites
*DS*: Down’s syndrome
*DSP*: Digital spatial profiling
*PA*: Pathological aging
*PART*: Primary aged-related tauopathy
*PSEN*: Presenilin
*MCI*: Mild cognitive impairment
*NFT*: Neurofibrillary tangles
*NP*: Neuritic plaques
*TANCs*: Tangle-associated neuritic clusters
*TREM2*: Triggering receptor expressed on myeloid cells 2
